# Sleep symptoms are essential features of long‐COVID – Comparing healthy controls with COVID‐19 cases of different severity in the international COVID sleep study (ICOSS‐II)

**DOI:** 10.1111/jsr.13754

**Published:** 2022-10-08

**Authors:** Ilona Merikanto, Yves Dauvilliers, Frances Chung, Yun Kwok Wing, Luigi De Gennaro, Brigitte Holzinger, Bjørn Bjorvatn, Charles M. Morin, Thomas Penzel, Christian Benedict, Adrijana Koscec Bjelajac, Ngan Yin Chan, Colin A. Espie, Harald Hrubos‐Strøm, Yuichi Inoue, Maria Korman, Anne‐Marie Landtblom, Damien Léger, Kentaro Matsui, Sergio Mota‐Rolim, Michael R. Nadorff, Giuseppe Plazzi, Catia Reis, Juliana Yordanova, Markku Partinen

**Affiliations:** ^1^ SleepWell Research Program, Faculty of Medicine University of Helsinki Helsinki Finland; ^2^ Department of Public Health and Welfare Finnish Institute for Health and Welfare Helsinki Finland; ^3^ Orton Orthopaedics Hospital Helsinki Finland; ^4^ Sleep‐Wake Disorders Center, Department of Neurology, Gui‐de‐Chauliac Hospital, Institute for Neurosciences of Montpellier INM, INSERM University of Montpellier Montpellier France; ^5^ Department of Anesthesiology and Pain Medicine University Health Network, University of Toronto Toronto Ontario Canada; ^6^ Li Chiu Kong Family Sleep Assessment Unit, Department of Psychiatry, Faculty of Medicine The Chinese University of Hong Kong Hong Kong China; ^7^ Department of Psychology Sapienza University of Rome Rome Italy; ^8^ IRCCS Fondazione Santa Lucia Rome Italy; ^9^ Institute for Consciousness and Dream Research Medical University of Vienna, Wien, Postgraduate Sleep Coaching Vienna Austria; ^10^ Department of Global Public Health and Primary Care University of Bergen Bergen Norway; ^11^ Norwegian Competence Center for Sleep Disorders Haukeland University Hospital Bergen Norway; ^12^ Centre de recherche CERVO/Brain Research Center, École de psychologie Université Laval Quebec City Quebec Canada; ^13^ Sleep Medicine Center Charite University Hospital Berlin Berlin Germany; ^14^ Department of Pharmaceutical Biosciences, Molecular Neuropharmacology Uppsala University Uppsala Sweden; ^15^ Institute for Medical Research and Occupational Health Zagreb Croatia; ^16^ Sleep and Circadian Neuroscience Institute, Nuffield Department of Clinical Neurosciences University of Oxford Oxford UK; ^17^ Department of Otorhinolaryngology Akershus University Hospital Lørenskog Norway; ^18^ Institute of Clinical Medicine University of Oslo Oslo Norway; ^19^ Department of Somnology Tokyo Medical University Tokyo Japan; ^20^ Japan Somnology Center Institute of Neuropsychiatry Tokyo Japan; ^21^ Department of Occupational Therapy, Faculty of Health Sciences Ariel University Ariel Israel; ^22^ Department of Medical Sciences, Neurology Uppsala University Uppsala Sweden; ^23^ Department of Biomedical and Clinical Sciences Linköping University Sweden; ^24^ Sleep and Vigilance Center Hopital Hotel‐Dieu de Paris Paris France; ^25^ VIFASOM (EA 7331 Vigilance Fatigue Sommeil et Santé Publique) Universite de Paris Paris France; ^26^ Department of Clinical Laboratory National Center Hospital, National Center of Neurology and Psychiatry Kodaira Japan; ^27^ Brain Institute, Physiology and Behavior Department Federal University of Rio Grande do Norte Natal Brazil; ^28^ Onofre Lopes University Hospital Federal University of Rio Grande do Norte Natal Brazil; ^29^ Department of Psychology Mississippi State University Mississippi State Mississippi USA; ^30^ IRCCS Istituto Delle Scienze Neurologiche di Bologna Bologna Italy; ^31^ Department of Biomedical, Metabolic and Neural Sciences University of Modena and Reggio Emilia Modena Italy; ^32^ Universidade Católica Portuguesa Católica Research Centre for Psychological—Family and Social Wellbeing Lisbon Portugal; ^33^ Instituto de Medicina Molecular João Lobo Antunes, Faculdade de Medicina de Lisboa Universidade de Lisboa Lisbon Portugal; ^34^ Instituto de Saúde Ambiental, Faculdade de Medicina Universidade de Lisboa Lisbon Portugal; ^35^ Institute of Neurobiology Bulgarian Academy of Sciences Sofia Bulgaria; ^36^ Department of Clinical Neurosciences, Clinicum University of Helsinki Helsinki Finland; ^37^ Helsinki Sleep Clinic Terveystalo Healthcare Helsinki Finland

**Keywords:** COVID‐19, excessive daytime sleepiness, fatigue, insomnia, pandemic, post‐acute sequelae of COVID‐19

## Abstract

Many people report suffering from post‐acute sequelae of COVID‐19 or “long‐COVID”, but there are still open questions on what actually constitutes long‐COVID and how prevalent it is. The current definition of post‐acute sequelae of COVID‐19 is based on voting using the Delphi‐method by the WHO post‐COVID‐19 working group. It emphasizes long‐lasting fatigue, shortness of breath and cognitive dysfunction as the core symptoms of post‐acute sequelae of COVID‐19. In this international survey study consisting of 13,628 subjects aged 18–99 years from 16 countries of Asia, Europe, North America and South America (May–Dec 2021), we show that post‐acute sequelae of COVID‐19 symptoms were more prevalent amongst the more severe COVID‐19 cases, i.e. those requiring hospitalisation for COVID‐19. We also found that long‐lasting sleep symptoms are at the core of post‐acute sequelae of COVID‐19 and associate with the COVID‐19 severity when COVID‐19 cases are compared with COVID‐negative cases. Specifically, fatigue (61.3%), insomnia symptoms (49.6%) and excessive daytime sleepiness (35.8%) were highly prevalent amongst respondents reporting long‐lasting symptoms after hospitalisation for COVID‐19. Understanding the importance of sleep‐related symptoms in post‐acute sequelae of COVID‐19 has a clinical relevance when diagnosing and treating long‐COVID.

## 1 INTRODUCTION

Post‐acute sequelae of COVID‐19 (PASC) represent an emerging global crisis. In the current WHO definition of PASC or “long‐COVID”, the three most common symptoms are fatigue, shortness of breath, and cognitive dysfunction/brain fog. Other reported symptoms are, for example, headache, post‐exertional malaise, muscle pains, cough and tachycardia. In the WHO definition, long‐lasting symptoms can also emerge after the initial recovery, and fluctuate or persist (Soriano et al., 2021). To date, studies on PASC in the general population are limited, and PASC is described based on either the Delphi‐method (Soriano et al., 2021) or hospital record data with no healthy control subjects (Ayoubkhani et al., 2021; Seeßle et al., 2022; Wong‐Chew et al., 2022). Therefore, it is crucial to distinguish which are the sequelae and other medical complications that last months after initial recovery from COVID‐19 (C‐19), and differentiate what constitutes PASC from the pandemic effects on general wellbeing. In order to delineate the symptom complex associated with PASC, there is a need for a “non‐infected” control population, as the pandemic has affected nearly everyone with an increase in physical and mental disturbances (Merikanto et al., 2022; Morin et al., 2011; Partinen et al., 2021). There is also a lack of studies examining differences in PASC symptoms by initial C‐19 severity (Lopez‐Leon et al., 2021). In particular, although sleep‐related problems in PASC have been reported, their importance in C‐19 has been understudied.

The main aim of the International COVID Sleep Study‐II in 2021 (ICOSS‐II) was to study the role of sleep and wake disturbances in PASC, as sleep problems have increased significantly during the pandemic (Merikanto et al., 2021; Partinen et al., 2021). Accordingly, we examined the prevalence and nature of sleep problems along with other previously defined PASC symptoms lasting at least 3 months in relation to C‐19 severity with non‐C‐19 controls.

## 2 METHODS

The ICOSS‐group conducted (May–Dec 2021) an international harmonised online survey in 16 countries (Austria, Brazil, Bulgaria, Canada, Hong Kong/China, Croatia, Finland, France, Germany, Israel, Italy, Japan, Norway, Portugal, Sweden, USA; Merikanto et al., 2021). The study conforms to recognised standards by the Declaration of Helsinki. All investigators obtained local ethical committee (REB) approval. However, due to the anonymous nature of the survey, REB permissions were exempted in some countries.

A total of 15,813 participants who responded to the survey gave their informed consent, and information on their gender and age. The analytic sample consists of 13,628 subjects of 18–99 years old (69.1% women, mean age of 42.7 years, SD = 16.6 years), and information on whether they have had C‐19 confirmed by C‐19 antigen/polymerase chain reaction (PCR) test as well as the severity of the C‐19. Those reporting having had C‐19 confirmed with a positive C‐19 antigen/PCR test were classified as C‐19 cases (*N* = 2705, 19.8%), while those without reported C‐19 and without a positive test were classified as non‐C‐19 controls (*N* = 10,923). The severity of the acute C‐19 was given in four groups: (1) asymptomatic (no marked symptoms during the acute C‐19; *N* = 295, 10.9% of the C‐19 cases); (2) mild (symptoms disappeared without specific medications; *N* = 1497, 55.3% of the C‐19 cases); (3) moderate (medications used, no severe pneumonia, no extra oxygen needed; *N* = 683, 25.2% of the C‐19 cases); and (4) severe/life‐threatening (hospitalisation with iv‐lines or intensive care unit [ICU] admission; *N* = 230, 8.5% of the C‐19 cases).

Complex sample procedures in IBM SPSS Statistics 27 were used in statistical analyses examining the prevalence of sleep problems along with other previously defined PASC symptoms lasting at least 3 months and demographic differences in relation to C‐19 severity as compared with non‐C‐19 controls. Analyses were weighted by giving a weight of equal share for each country, and by the joint distribution of age and gender of each participating country/area.

## 3 RESULTS

### 3.1 Differences in demographics, concomitant long‐lasting symptoms and vaccination status between non‐C‐19 controls and different severity of C‐19

As presented in Table 1, severe/life‐threatening C‐19 was more common amongst men than amongst women as compared with non‐C‐19 controls and milder severity C‐19 cases (*p* < 0.05). Severe/life‐threatening C‐19 cases were older (*p* < 0.001) and had higher body mass index (BMI; *p* < 0.001) with more obesity (*p* < 0.001) than non‐C‐19 controls and milder severity C‐19 cases. Non‐C‐19 controls and milder severity C‐19 cases were more often single than in a relationship (*p* < 0.001), and were students or in regular day work while shift/night work. Temporarily laid‐off or retired were more common amongst severe/life‐threatening C‐19 cases (*p* < 0.001). Higher education was less common amongst severe/life‐threatening C‐19 cases than amongst non‐C‐19 controls and milder C‐19 cases (*p* < 0.001).

**TABLE 1:** Differences in demographics, concomitant long‐lasting symptoms and vaccination status between non‐C‐19 controls and different severity of C‐19

	Non‐C‐19 controls	Asymptomatic C‐19 cases	Mild C‐19 cases	Moderate C‐19 cases	Severe/life‐threatening C‐19 cases	*p*‐Value
***Demographics***						
**Gender (%)**						
Man	47.6	49.9	44.2	43.3	57.9	<0.05
Woman	52.4	50.1	55.8	56.7	42.1	
**Age (Mean, SE)**	47.2 (0.3)	46.6 (1.7)	40.7 (0.7)	47.8 (1.0)	58.1 (1.1)	<0.001
**BMI (Mean, SE)**	25.7 (0.1)	25.9 (0.6)	26.1 (0.3)	26.8 (0.4)	29.2 (0.5)	<0.001
**BMI above 30 (%)**						<0.001
No	83.6	87.7	84.1	75.9	64.5	
Yes	16.4	12.3	15.9	24.1	35.5	
**Marital status (%)**						<0.001
Single	28.2	27.9	37.0	31.1	14.3	
Married/relationship	60.3	54.8	57.3	59.5	75.2	
Divorced or separated	8.7	7.6	4.2	5.5	7.7	
Widowed	2.7	9.6	1.5	3.9	2.8	
**Highest level of education (%)**						
Primary/elementary/lower secondary school	4.1	4.3	4.8	4.4	12.4	<0.01
Secondary school/high school/vocational school	32.0	35.0	26.6	25.9	42.5	
University, College or above	63.9	60.7	68.6	69.7	45.1	
**Present work (%)**						<0.001
Student	14.2	18.1	21.1	14.4	5.5	
Regular day work	45.1	45.9	47.2	50.3	31.0	
Irregular day work/freelancer/artist/research	8.4	9.7	9.9	9.2	8.8	
Shift work/night work	4.2	1.0	6.9	4.6	12.7	
Unemployed	3.1	4.9	3.9	6.8	4.4	
Retired	20.4	19.0	8.3	13.1	34.7	
At home (no salary)	4.0	1.2	1.8	1.2	0.5	
Temporarily laid off	0.6	0.1	0.8	0.3	2.3	
***Concomitant symptoms***						
**Concomitant long‐lasting symptoms (Mean, SE)**	1.7 (0.04)	1.6 (0.2)	2.9 (0.1)	4.6 (0.3)	6.1 (0.5)	<0.001
***Vaccination status***						
**Vaccinated against C‐19 (%)**						<0.001
No	14.9	27.7	33.6	34.8	34.4	
One vaccination	15.1	36.6	31.1	29.0	36.4	
Two vaccinations	70.0	35.7	35.3	36.2	29.1	
***Diagnosed mental disorders***						
Depression						<0.001
Do not have	83.2	88.0	81.9	73.9	71.6	
Have had it already before the pandemic	13.2	5.7	10.7	12.1	9.9	
Developed depression during the pandemic without relation to infection	3.5	4.7	2.8	2.7	1.5	
Developed this after I had a C‐19 infection	0.1	1.7	4.5	11.2	16.9	

*Note*: *p*‐Values are based on chi‐square tests within SPSS complex samples crosstabs or *t*‐tests within SPSS complex samples general linear model (CSGLM). BMI, body mass index; C‐19, COVID‐19.

There was a dose‐dependent response with more concomitant long‐lasting symptoms for at least 3 months amongst more severe C‐19 cases than amongst milder cases (*p* < 0.001). Non‐C‐19 controls and asymptomatic cases both had less than two concomitant long‐lasting symptoms, while there were on average four–six concomitant symptoms amongst mild to severe C‐19 cases, respectively.

Severe/life‐threatening C‐19 cases were less vaccinated against C‐19 than milder C‐19 cases, while the level of vaccination was higher amongst the non‐C‐19 controls (*p* < 0.001). Lastly, severe/life‐threatening C‐19 cases reported significantly more depression developed after the infection, than milder severity C‐19 cases (*p* < 0.001).

### 3.2 Long‐lasting PASC symptoms in relation to C‐19 severity

**FIGURE 1:**
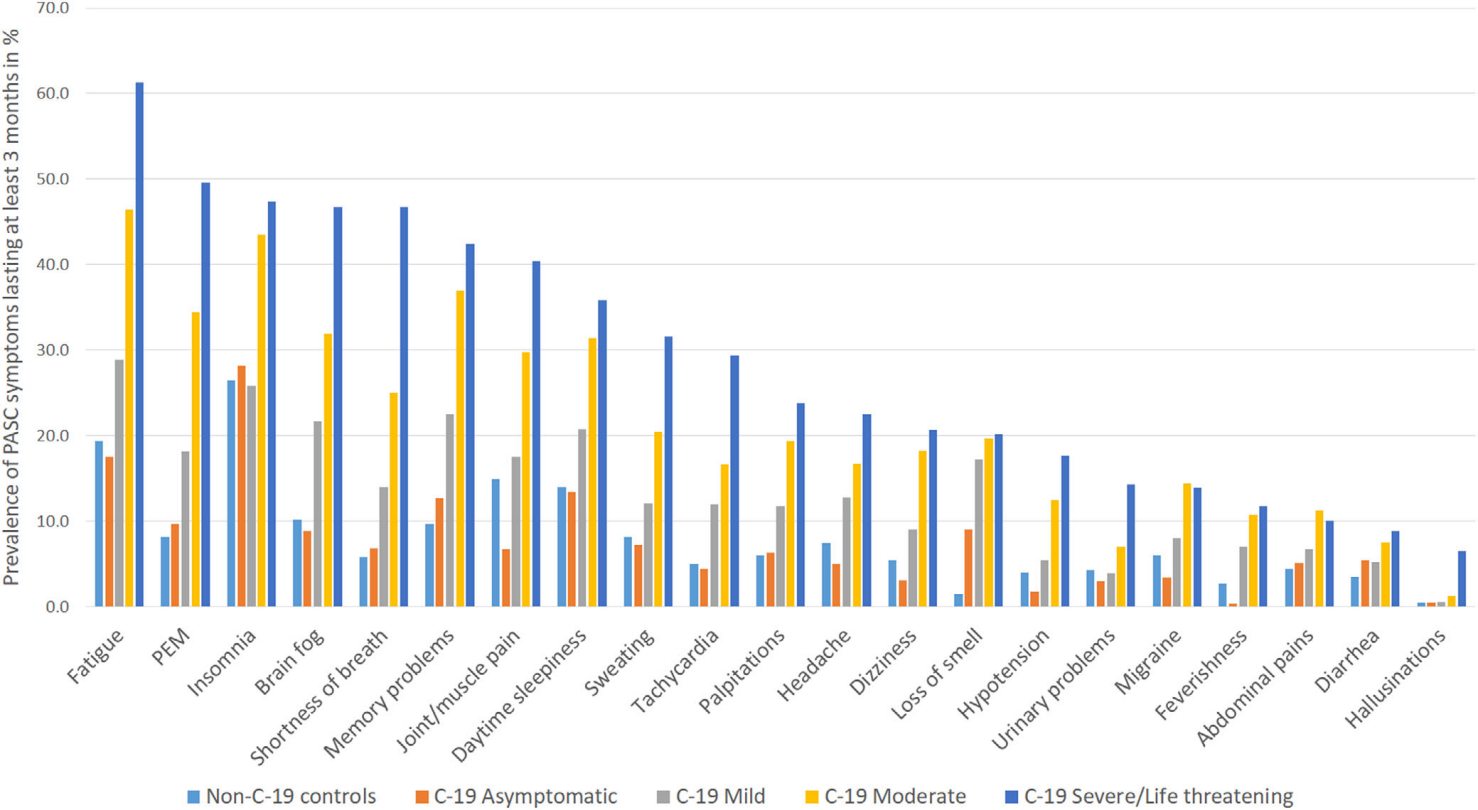
Occurrence of long‐COVID (post‐acute sequelae of COVID‐19; PASC) symptoms lasting for at least 3 months in percentage amongst non‐COVID (C‐19) controls and in different C‐19 groups with different severity. *Abdominal pain*: “Abdominal pains, colics”; *Brain fog*: “Problems of attention or of concentration and/or brain fog or cognitive dysfunction”; *Flu‐like symptoms*: “Feverishness and/or flu‐like symptoms such as sore throat, runny nose, etc.”; *Hallucinations*: “Hallucinations, psychotic symptoms”; *Headache*: “Migraine or headache, other than migraine”; *Heart palpitations*: “Palpitations and/or cardiac arrhythmia”; *Insomnia*: “Difficulties falling or staying asleep”; *Joint/muscle pain*: “Joint pain (arthralgia) and/or muscle pain, muscle aches”; *Loss of smell*: “Loss of smell and/or taste”; *Low blood pressure*: “Low blood pressure (hypotension)”; *Nausea, diarrhoea*: “Nausea and/or diarrhoea or vomiting”; *Orthostatic dizziness*: “Dizziness when standing”; *PEM* : “Post‐exertional malaise referring to prolonged weakness/poor functioning after exertion, such as muscle weakness, difficulties walking long distances”; *Shortness of breath*: “Shortness of breath or difficulty breathing and/or chest pain”; *Sleepiness*: “Excessive daytime sleepiness”; *Sweating problems*: “Problems of sweating and/or trouble tolerating cold/heat”; *Tachycardia*: “Tachycardia, fast pulse rate”

The occurrence of PASC symptoms relative to the severity of C‐19 is shown in Figure 1. The prevalence of all the 21 PASC symptoms differed significantly between the C‐19 controls and cases in Complex Survey chi‐square tests (all *p*‐values < 0.001). Figure 1 shows that the prevalence of most of the long‐lasting symptoms was lower amongst non‐C‐19 controls and asymptomatic cases than amongst mild C‐19 cases, with moderate and severe cases having the highest prevalence of the long‐lasting symptoms.

Almost all of the PASC symptoms were most common in the severe/life‐threatening cases. Of the 21 symptoms amongst C‐19 cases, fatigue, insomnia and excessive daytime sleepiness were amongst the eight most common PASC symptoms regardless of the disease severity (Figure 1). In general, the eight most common long‐lasting symptoms amongst C‐19 cases amongst severe/life‐threatening C‐19 cases were: fatigue (61.3%); post‐exertional malaise (PEM; 49.6%); insomnia (47.4%); brain fog (46.7%); shortness of breath (46.7%); problems of memory (42.4%); joint/muscle pains (40.4%); and excessive daytime sleepiness (35.8%). The prevalence of joint/muscle pains and shortness of breath was not as common as the other six most common PASC symptoms amongst the moderate C‐19 cases. When comparing non‐C‐19 controls and asymptomatic cases, long‐lasting loss of smell was far more common amongst asymptomatic cases (9.1%) than amongst non‐C‐19 controls (1.4%), although not as common as amongst more severe C‐19 cases (17.2%–20.1%, respectively). On the other hand, long‐lasting joint/muscle pain was more common amongst non‐C‐19 controls (14.9%) than amongst asymptomatic cases (6.7%).

## 4 DISCUSSION

We observed a high frequency of long‐lasting sleep disturbances characterised by difficulty falling asleep or staying asleep and excessive daytime sleepiness in C‐19 cases, which contrasts with previous studies that have failed to assess the frequency of sleep symptoms in PASC. PASC symptoms were similar amongst moderate and severe C‐19 cases, although more common amongst the more severe cases. Shortness of breath was emphasised amongst the severe/life‐threatening C‐19 cases as compared with moderate cases as one of the five most common PASC symptoms. Amongst moderate cases, it was the eighth most common PASC symptom out of 21 potential PASC symptoms. This is understandable, as patients treated in an ICU may have more severe pulmonary pathology (Ayoubkhani et al., 2021).

Our finding that fatigue, cognitive problems and shortness of breath are amongst the most common long‐lasting symptoms in hospitalised C‐19 patients fits with previous reports (Lopez‐Leon et al., 2021). Especially fatigue and cognitive dysfunction are symptoms previously related to other post‐viral conditions, such as post‐viral fatigue syndrome (Komaroff & Bateman, 2021). A previous study from Wong et al. (Wong‐Chew et al., 2022) that compared hospitalised C‐19 patients who had or had not received invasive mechanical ventilation showed a similar trend of higher prevalence of PASC symptoms amongst more severe C‐19 cases, as with our findings. This difference disappeared after 3 months of discharge, and comparisons were made only between hospitalised patients (Wong‐Chew et al., 2022), and not between severe cases and milder or asymptomatic cases as we have done.

Previous studies have not assessed sleep‐related long‐lasting symptoms, and have failed to include non‐C‐19 controls to compare the impact posed to health by PASC versus the general effects of the pandemic. Our findings indicate that fatigue, insomnia symptoms and excessive daytime sleepiness are amongst the most common symptoms of PASC, which have not been considered in the previous criteria of PASC (Lopez‐Leon et al., 2021; Soriano et al., 2021). Furthermore, all the long‐lasting symptoms we examined were emphasised amongst C‐19 cases and especially amongst the more severe cases as compared with non‐C‐19 controls, indicating that they are linked to PASC.

Our findings suggest that severe/life‐threatening C‐19 cases are more prone to more complex PASC with a higher prevalence of concomitant symptoms than those with milder C‐19. PASC has previously been also associated with worsened mental health (Malik et al., 2022). Our findings support this, indicating that especially severe/life‐threatening C‐19 cases report more depression developed after C‐19 infection. The high prevalence of long‐lasting complex concomitant symptoms can create a vicious circle between physical and mental symptoms that elevate the risk for depression and chronic conditions. For instance, the bidirectional relation between depression and sleep problems is well established (Fang et al., 2019).

Severe/life‐threatening C‐19 cases in our sample reported more risk factors previously indicated for severe C‐19, such as a higher prevalence of BMI greater than 30 kg m^−2^ (Li et al., 2021). Severe/life‐threatening C‐19 cases were on average older than 55 years, and more often of male gender than milder C‐19 cases, features that have also been previously indicated as risk factors for more severe C‐19 (Gallo Marin et al., 2021; Li et al., 2021). We found that the severe C‐19 cases were on average less educated, more often in a relationship, and in shift/night work, temporarily laid‐off or retired. Differences in behavioural, socioeconomic and age‐related factors between the groups can play a role in the risk of encountering the virus and how equipped the body is to fight against the viral effects. A very clear difference between the non‐C‐19 controls and the C‐19 cases was seen in the level of vaccination, with non‐C‐19 controls being significantly more vaccinated and the more severe C‐19 cases least vaccinated in line with the evidence on vaccines being a powerful tool in reducing the risk for infection and hospitalisation (Mohammed et al., 2022).

The strengths of our study include the international sample targeting the general population, allowing comparison of different C‐19 cases to non‐C‐19 controls to separate the psychological effects of the pandemic from the C‐19‐induced effects on the occurrence of long‐lasting symptoms. Limitation of the survey‐based data is the reliability of self‐reported information as compared with objectively measured data.

## 5 CONCLUSION

We recommend that sleep symptoms including both daytime sleepiness and insomnia symptoms be more systematically assessed when evaluating patients with PASC/long‐COVID. Diagnosis, prevention and treatment of PASC require holistic approaches, with the need for multi‐disciplinary teams including sleep specialists to optimise the treatment of patients with PASC.

## AUTHOR CONTRIBUTIONS

Writing the original manuscript and statistical analyses by Ilona Merikanto and Markku Partinen. Study design by the ICOSS second survey core group: Ilona Merikanto, Frances Chung, Yves Dauvilliers, Luigi De Gennaro, Brigitte Holzinger, Yun Kwok Wing, Markku Partinen. All authors participated in data collections and approved the final manuscript version.

## FUNDING INFORMATION

This study was funded by The Academy of Finland (project 322312, recipient Dr Merikanto), The Finnish Cultural Foundation (recipient Dr Merikanto), and the Signe and Ane Gyllenberg Foundation (recipients Prof. Partinen and Dr Merikanto). The funders had no role in study design, data collection and interpretation, or the decision to submit the work for publication. There are no competing financial interests. Financial disclosure: none. Non‐financial disclosure: none.

## CONFLICT OF INTEREST

None declared.

## Data Availability

Data from each country are owned by the ICOSS principal investigators of that country but for research purposes the data from different countries can be pooled together and accessed freely by other researchers involved in the ICOSS collaboration. Study plans are to be accepted by the ICOSS second survey core group.
